# Empowering Health and Identity: The Vital Role of Gender-affirming Care for Individuals With HIV

**DOI:** 10.1093/ofid/ofae435

**Published:** 2024-07-23

**Authors:** Lauren Touleyrou, Gretchen Newman, Shira Heisler, Teena Chopra

**Affiliations:** Internal Medicine/Infectious Diseases, Wayne State University, Detroit, Michigan, USA; Internal Medicine/Infectious Diseases, Wayne State University, Detroit, Michigan, USA; Internal Medicine/Infectious Diseases, Wayne State University, Detroit, Michigan, USA; Internal Medicine/Infectious Diseases, Wayne State University, Detroit, Michigan, USA

**Keywords:** ART, gender-affirming hormone therapy, HIV, transgender

## Abstract

Comprehensive healthcare for all includes gender-affirming hormone therapy for transgender and nonbinary individuals. It is the unique privilege of HIV providers, who take care of a disproportionate number of transgender people, to provide gender-affirming hormone therapy along with antiretroviral therapy. It could increase viral suppression rates, increase overall health outcomes, and decrease gender health disparities.

Recent data from the Center for Disease Control and Prevention's Behavior Risk Factor Surveillance System estimates that more than 5.5% of the United States identify as lesbian, gay, bisexual, transgender, queer/questioning (LGBTQIA+) [1]. A total of 1.3 million adults identify as transgender. More than 4% of the Detroit population identify as LGBTQIA+ [2]. As we strive to ensure comprehensive healthcare for all, it is imperative to address the intersecting needs of transgender and nonbinary individuals who face disproportionate challenges accessing essential medical services, including HIV care. A recent study found that 38% of LGBTQ+ people in Michigan had a negative interaction with a healthcare provider related to being transgender [3]. This included being denied treatment, verbally harassed, assaulted, or having to teach the provider about transgender people to receive appropriate care.

The estimated prevalence of HIV among transgender woman and transgender men is 42.0% and 3.2% respectively [4]. Moreover, transgender people of color bear a disproportionate burden, with 51% of transgender women and 58% of transgender men living with HIV identifying as Black or African American [5]. In Detroit alone, the impact is stark, with more than 189 male-to-female transgender individuals living with HIV as of 2021, constituting about 2.2% of new HIV diagnoses annually [6, 7]. However, it is crucial to note that these figures likely underestimate the true number of transgender individuals living with HIV because gender identity data was not systematically collected before 2010 by Michigan Department of Health and Human Services.

Transgender women living with HIV have poorer outcomes among the entire HIV care cascade, including lower retention to care, use of antiretroviral therapy (ART), adherence to ART, and viral suppression [8]. The enduring stigma surrounding HIV and accessing HIV care, coupled with the additional stigma experienced by transgender individuals, underscores the critical importance of HIV providers delivering comprehensive gender-affirming care alongside prescribing ART. By addressing both HIV-related and gender-specific healthcare needs, providers can better support the holistic well-being and health outcomes of transgender individuals with HIV.

The prescription of gender-affirming hormone therapy along with ART is imperative. Coprescription could increase retention in care, adherence to ART, and viral suppression. A study based out of a Ryan White–funded HIV clinic in Tennessee found that there was increased retention in care and maintenance of viral suppression, though, not statistically significant [9]. It is vital that this care is coprovided during routine HIV visits with care providers. Because access to medical care is a challenge in Detroit, it is imperative that all medical care that can be provided under 1 roof should be. To ensure that HIV specialty clinics provide equitable care to LGBTQ+ patients, providers should feel comfortable providing gender-affirming hormone therapy.

A study from 2014 out of San Francisco echoed similar sentiments as listed previously. Focus groups and individual interviews were conducted with 20 transgender women living with HIV focusing on experiences with testing, diagnosis, and treatment [10]. Participants prioritized obtaining hormone therapy over ART care but stated that if the medications were available from the same provider, they would be more likely to be adherent to ART and be maintained in HIV care.

In conclusion, by integrating gender-affirming care with HIV treatment, participating in initiatives like the Healthcare Equality Index, and ensuring our providers are equipped to meet the diverse needs of LGBTQ+ patients, HIV providers can continue to lead in providing equitable and inclusive HIV care. Together, these efforts not only validate our commitment to serving all patients with dignity and respect but also reaffirm our role as HIV providers to comprehensive care for the LGBTQIA+ community.

## Note

***Potential conflicts of interest.*** All authors: No reported conflicts.
